# Long-term changes in cognitive bias and coping response as a result of chronic unpredictable stress during adolescence

**DOI:** 10.3389/fnhum.2013.00328

**Published:** 2013-07-04

**Authors:** Lauren E. Chaby, Sonia A. Cavigelli, Amanda White, Kayllie Wang, Victoria A. Braithwaite

**Affiliations:** ^1^Center for Brain, Behavior, and Cognition, Pennsylvania State University, University ParkPA, USA; ^2^Department of Ecosystem Science and Management, Pennsylvania State University, University ParkPA, USA; ^3^Department of Biobehavioral Health, Pennsylvania State University, University ParkPA, USA; ^4^Department of Biology, Pennsylvania State University, University ParkPA, USA

**Keywords:** adolescence, cognitive bias, coping, decision-making, chronic unpredictable stress, *Rattus norvegicus*, successive negative contrast

## Abstract

Animals that experience adverse events in early life often have life-long changes to their physiology and behavior. Long-term effects of stress during early life have been studied extensively, but less attention has been given to the consequences of negative experiences solely during the adolescent phase. Adolescence is a particularly sensitive period of life when regulation of the glucocorticoid “stress” hormone response matures and specific regions in the brain undergo considerable change. Aversive experiences during this time might, therefore, be expected to generate long-term consequences for the adult phenotype. Here we investigated the long-term effects of exposure to chronic unpredictable stress during adolescence on adult decision-making, coping response, cognitive bias, and exploratory behavior in rats. Rats exposed to chronic unpredictable stress (e.g., isolation, crowding, cage tilt) were compared to control animals that were maintained in standard, predictable conditions throughout development. Unpredictable stress during adolescence resulted in a suite of long-term behavioral and cognitive changes including a negative cognitive bias [*F*_(1, 12)_ = 5.000, *P* < 0.05], altered coping response [*T*_(1, 14)_ = 2.216, *P* = 0.04], and accelerated decision-making [*T*_(1, 14)_ = 3.245, *P* = 0.01]. Exposure to chronic stress during adolescence also caused a short-term increase in boldness behaviors; in a novel object test 15 days after the last stressor, animals exposed to chronic unpredictable stress had decreased latencies to leave a familiar shelter and approach a novel object [*T*_(1, 14)_ = 2.240, *P* = 0.04; *T*_(1, 14)_ = 2.419, *P* = 0.03, respectively]. The results showed that stress during adolescence has long-term impacts on behavior and cognition that affect the interpretation of ambiguous stimuli, behavioral response to adverse events, and how animals make decisions.

## Introduction

Negative life experiences can have long-term effects on behavior and physiology (Sheriff et al., 2009; Archard et al., 2012). Stressful events (e.g., stressors) come in a variety of forms, but in vertebrates they are often considered to be unpredictable aversive stimuli that provoke a glucocorticoid hormone response mediated by the hypothalamic-pituitary-adrenal (HPA) axis (Spear, 2000; Koolhaas et al., 2011). Stages of development differ in sensitivity to stress, certain life stages have specific vulnerabilities that can lead to different, permanent changes in future responses to adverse events (McCormick and Mathews, 2008; Vidal et al., 2011). For example, in zebra finches (*Taeniopygia guttata)* exposure to excess heat during early-life enables the birds to modify their response to subsequent heat exposures in adulthood to minimize oxidative damage (Costantini et al., 2012). Similarly, rodent pups that experience isolation at different stages of development exhibit contrasting hormonal responses to stress in adulthood; rat pups separated from their mothers for 2 h a day at postnatal days 2–14 develop a hyper-responsive HPA axis, whereas pups isolated at postnatal days 15–16 develop a hypo-functioning HPA axis (Plotsky and Meaney, 1993; Sánchez et al., 1998; reviewed in Sánchez et al., 2001).

During the adolescent stage, glucocorticoid production in response to a stressor exceeds the adult hormone response in duration and intensity (McCormick et al., 2010). In comparison to adult rats, adolescent rats exposed to an acute stressor show a higher increase in both adrencorticotrophin hormone (ACTH) and glucocorticoids (reviewed in Romeo and McEwen, 2007; Foilb et al., 2011). Additionally, during adolescence various neural structures involved in stress and reward processing are still immature (Spear, 2000; McCormick and Mathews, 2008). These characteristics of the adolescent life stage suggests that this period may be particularly vulnerable to effects from chronic glucocorticoid exposure (Romeo and McEwen, 2007; McCormick et al., 2010). Chronic exposure to elevated levels of glucocorticoid hormones has numerous effects on the brain including suppressed neurogenesis and enhanced dendritic pruning in the hippocampus, dendritic shortening in the medial prefrontal cortex, and enhanced dendritic growth in the amygdala, the fear center of the brain (reviewed in McEwen, 2005).

Adverse experiences during adolescence can impact the maturation of the central nervous system, shape future reward responses, and influence endocrine and behavioral function in adulthood (Romeo, 2003; McCormick et al., 2004; Andersen and Teicher, 2008; McCormick and Green, 2012). The changes that occur following stress exposure during adolescence are dynamic; some are immediate, some are short in duration, and some are long-term but only become apparent after an acute stressor is applied (McCormick and Green, 2012; McCormick et al., 2012; Saul et al., 2012). McCormick et al. (2012) found that exposure to unpredictable social instability and isolation in adolescent rats resulted in learning deficits in adulthood, but these effects were only apparent after an acute stressor was applied (McCormick et al., 2012). Others have reported that behavioral effects of stress during adolescence can be transient and fade over the lifetime of an animal (e.g., unpredictable isolation and novel social partner stressors during adolescence induce temporary changes in boldness, Mathews et al., 2008). Despite these important early studies, the long-term effects of stress during adolescence on emotion and cognition are not well-characterized. Yet, if we are to understand how animals cope with stress during development, and how early adverse experiences can prepare an animal to deal with subsequent stressors, we need to determine the long-term impacts of stress during the adolescent stage (Romeo, 2010).

A number of studies have demonstrated that stress during adolescence, including unpredictable chronic social and physical stress, can impact HPA axis function and glucocorticoid production in adulthood (McCormick and Mathews, 2008; Buwalda et al., 2011). The long-term consequences of stress during adolescence on cognition and behavioral coping response, however, remains unknown. A method to assess behavioral coping response and reward loss sensitivity, as mediated by glucocorticoid production, is the successive negative contrast (SNC) test (Mitchell and Flaherty, 1998; Gomez et al., 2009). SNC has been used for over 3 decades to evaluate an animal's response to the unexpected downshift of a familiar high-value reward to a novel low-value reward (Lombardi and Flaherty, 1978; Flaherty and Rowan, 1989). Recently SNC has been used as a measure of coping response to infer background emotional state in non-human animals (Burman et al., 2008; Gomez et al., 2009).

In humans, background emotional state can affect decision-making through a cognitive bias in stimulus interpretation that impacts stimulus perception, attention, and processing (Winkielman et al., 2007). Increasingly, measures of cognitive bias are being used as indicators of background emotional state in non-human animals (Burman et al., 2009; Brydges et al., 2011). Unlike most behavioral and physiological measures, cognitive bias tests can measure the valence of affect (positivity vs. negativity) rather than just arousal (Mendl and Paul, 2004). Prior studies have shown that adult rats can exhibit a negative cognitive bias, marked by an increased propensity to interpret ambiguous stimuli as threatening or aversive that can start during stress exposure and last up to several days after an aversive events (Harding et al., 2004; Burman et al., 2009). The potential longevity of a negative cognitive bias following exposure to stress, however, remains unclear (Mendl et al., 2009; but see Brydges et al., 2012). A previous focus on short-term changes in cognitive bias has meant that long-term changes have so far been underexplored (Brilot et al., 2010). In the current study, we addressed the long-term effects of chronic unpredictable stress during adolescence on behavior and cognition by evaluating changes in cognitive bias, decision-making, associative learning rate, coping response, and motivation to consume a reward in adulthood.

A range of behavioral tests were used to examine the consequences of stress during adolescence: (1) sucrose preference (Strekalova et al., 2004), (2) exploration of a novel object (Van Dijken et al., 1992; Cavigelli et al., 2009), (3) successive negative contrast (SNC), and (4) ambiguous judgment cognitive bias (Harding et al., 2004; Doyle et al., 2011). We measured exploratory behavior and motivation to consume a reward because alterations in these fundamental traits could potentially affect the interpretation of more complex reward or activity based tests including the cognitive bias and SNC tests. Stress can alter both the motivation to consume a reward and exploratory behavior; the magnitude of effects from stress are dependent upon the type and duration of the stressors and traits intrinsic to the animal (Zurita et al., 2000; Strekalova et al., 2004; Brilot et al., 2010). We hypothesized that stress during adolescence would induce a negative cognitive bias and stronger sensitivity to reward loss, both suggestive of a long-term negative background emotional state. Additionally, we predicted that stress during adolescence would result in altered decision-making, impaired associative learning, and decreased exploratory behavior in adulthood.

## Methods

### Subjects and housing

**Figure 1:**
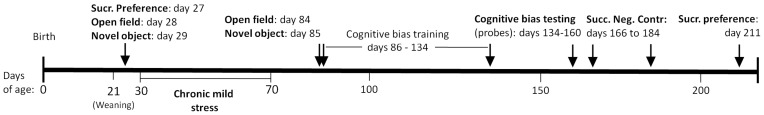
**Timeline of procedures**. We evaluated the long-term effects of chronic unpredictable stress during the adolescent life stage on adult cognition and behavior using the following tests: cognitive bias, successive negative contrast, sucrose preference, novel object, and open field.

Sixteen male Long-Evans rats (Harlan Laboratory in Fredrick, Maryland, USA) were obtained at 21 days of age. Following transport, rats were given 7 days to settle before handling and behavioral testing commenced. A full timeline of all manipulations and behavioral tests is provided in Figure 1. Animals were pair-housed in plastic cages, 20 × 26 × 46 cm, with corn cob bedding and basic enrichment items: two 7.6 cm diameter PVC tubes hanging from the wire cage lid and two 2.5 × 2.5 × 8 cm pine wood blocks. Rats were kept on a 12:12 reversed light/dark cycle at 20–21°C and 41–42% relative humidity. Standard rat chow (LabDiet®) and tap water were available *ad-libitum* unless otherwise noted. To minimize disturbance, the experimenter was not in the room during data collection. Work was approved by the Pennsylvania State University IACUC committee, protocol #35761.

### Adolescent chronic unpredictable stress

Four cages of pair housed rats (*n* = 8) were randomly assigned to the control condition and four cages (*n* = 8) to the stress treatment. For the latter group, stressors were presented daily from 30 to 70 days of age, with 8 days of rest occurring intermittently. Prior studies of adolescent-stress have varied in the duration of stress exposure, due in part to the large window of time during which adolescent ontogenetic changes occur. These changes are thought to conclude at approximately 55–60 days of age in male rodents (Spear, 2000). To cover the entirety of the ontogenetic window of adolescence, studies have included a postpubertal “sub-adult” period (Schmidt et al., 2007). Studies of adolescent-stress have used stress exposure periods spanning from 28 to 80 days of age (Spear, 2000; Sterlemann et al., 2010). As the current study evaluated behaviors mediated by the prefrontal cortex (i.e., decision-making, coping), and this region is still developing in early adulthood, the duration of stress exposure (30–70 days of age) included a postpubertal period in early adulthood (van Eden et al., 1990; Spear, 2000).

For the chronic unpredictable stress procedure both physical and social stressors were presented randomly across the light/dark cycle to maximize unpredictability. An average of three physical and three social stressors were presented between each rest day. Stressors noted to induce short-term changes in cognitive bias were used (e.g., cage tilt, damp bedding: Harding et al., 2004; e.g., crowding, confinement: Doyle et al., 2011; see Table 1). An additional stressor, isolation, was chosen because it has been associated with long-term changes in behavior following exposure during adolescence (McCormick et al., 2012).

**Table 1:** **Chronic unpredictable stressor descriptions**.

**PHYSICAL**	
Smaller cage	Rat pairs were housed for 4 h in a cage with a 25% reduction in volume from the 20 × 26 × 46 cm standard home cage (Doyle et al., 2011).
Damp bedding	While rats were temporarily in an empty transfer cage, 200 ml of water was mixed into 2/3 of the bedding of the home cage. After 6 h in the damp bedding, pairs were transferred to a clean home cage (Zurita et al., 2000; Harding et al., 2004).
Cage tilt	Home cages were tilted at a 30° angle for 6 h (Zurita et al., 2000; Harding et al., 2004).
**SOCIAL**	
Isolation	Rats were housed individually for 1.5 h in a clean cage (20 × 26 × 46 cm) with a 7.6 cm diameter PVC tube and a 2.5 × 2.5 × 8 cm pine wood block (Zurita et al., 2000; McCormick et al., 2012).
Crowding	Sets of 2 rat pairs were combined into one clean cage (20 × 45 cm) for 4 h; iterations of pair combinations were balanced (Zurita et al., 2000; Harding et al., 2004; Doyle et al., 2011).
Foreign bedding	Experimental pairs were housed in the empty home cage of a pair of older conspecifics for 12 h. (Harding et al., 2004).

To control for the influence of circulating corticosterone on tests mediated by glucocorticoid levels, such as the SNC, we controlled for daily rhythms in glucocorticoid production by avoiding testing during peak corticosterone production; all tests started a minimum of 2 h after the beginning of the dark cycle and were completed within 6 h of the start of the test (Mitchell and Flaherty, 1998). Weight and physical appearance were monitored; no changes in aggression or health related to either the unpredictable stress regimen or any of the behavioral tests were observed.

### Successive negative contrast (SNC)

**Figure 2:**
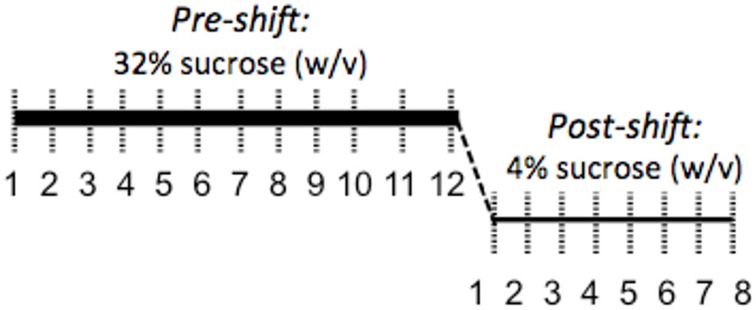
**Illustration of the SNC procedure to assess coping response**. A high-value 32% sucrose reward was presented for 5 min per day for 12 days while licks were counted to evaluate motivation to consume a reward. Following this 12-day acclimation period, the reward was unexpectedly decreased to 4% sucrose. The resulting decrease in consumption of the 4% solution is interpreted as a negative reaction to the violation of positive expectations and a measure of coping response.

Coping response was evaluated from 166 to 184 days of age with an SNC test measuring response to an unexpected downshift in reward value (Burman et al., 2008; Gomez et al., 2009: see Figure 2). During the SNC test, individual animals were tested daily in an opaque, plastic container, 30.5 × 30.5 × 30.5 cm, for 5 min. A plastic bottle of sucrose solution was attached to the center of one wall. Motivation to consume sucrose solution was measured with a basic electronic device attached to a computer that registered each lick through the closing of a circuit, the computer then provided a record of licking rates. After an initial 12 days of trials with a 32% sucrose (w/v) reward, the solution concentration was decreased without warning to 4% (w/v). The lower concentration was administered for 7 days to monitor the recovery of lick rates. To ensure reward salience, 2 h of food deprivation preceded each trial. We defined animals as having learned the SNC task upon registering 10 licks in one session; pre-shift data were evaluated from the first day that more than 60% of the animals had learned the task (day 4) to the last day of 32% sucrose solution presentation (day 12).

### Cognitive bias, decision-making, and associative learning

**Figure 3:**
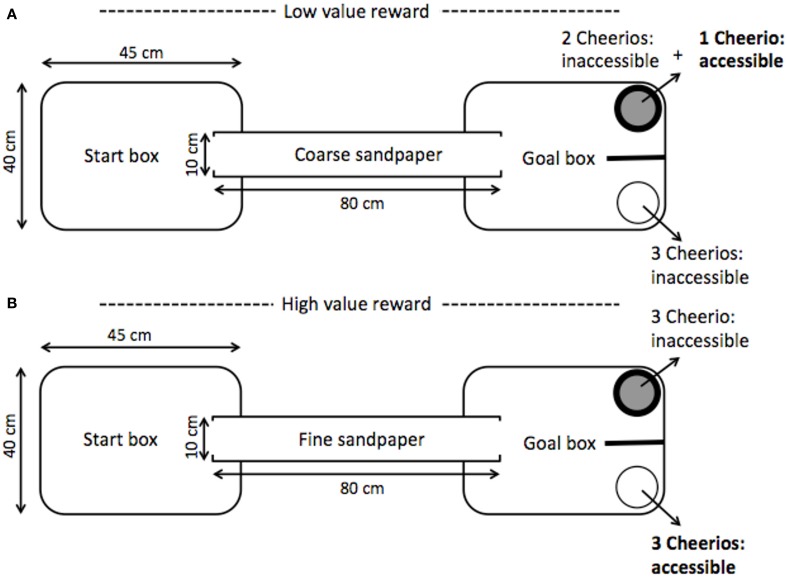
**Schematic of cognitive bias testing chamber adapted from Brydges et al. (2011)**. Within a daily session, 2 trials of each example panel were presented. (Panel **A**) depicts a low-value reward trial; the coarse sandpaper provided a cue that the low-value (1 Cheerio) reward was accessible whereas the fine sandpaper indicated a high-value reward (3 Cheerios) was present (Panel **B**). Sandpaper grade, bowl color, bowl side, and reward value pairings were counterbalanced. To balance scent cues from the rewarded and unrewarded bowls inaccessible Cheerios were present beneath a mesh barrier. After passing a learning criterion, animals were presented with a novel cue ambiguous in its equal distance from the two trained sandpaper cues. To indicate an interpretation of the ambiguous cue as closer to either a high or low-value reward the animal moved to either the high or low-reward location in the testing chamber. Interpretation of the ambiguous paper as closer to either the cue for the high or low-value reward conveyed a positive or negative value assignment from properties intrinsic to the animal, i.e., a positive or negative cognitive bias in the interpretation of ambiguity.

The ambiguous judgment task was used to assess the long-term impacts of stress during adolescence on cognitive bias, decision-making, and associative learning. Using a paradigm similar to Brydges et al. (2011), animals were trained to associate a conditioned stimulus, a type of sandpaper (rough or smooth), with the location and color of a bowl containing an available food reward. To do this, individuals were placed in a 30 × 40 × 45 cm opaque plastic start box that was connected to a goal box by an 80 cm PVC pipe (see Figure 3). The goal box contained a white bowl and a black bowl separated by an opaque partition to ensure that the rats made a choice between the two bowls upon exiting the PVC tunnel. Of the two available bowls, one was associated with a high-value reward (3 Cheerios), the other with a low-value reward (1 Cheerio). To balance the scent cues, each bowl always contained three Cheerios, but the accessibility of the rewards varied depending on the trial condition. For a high-reward trial, 3 Cheerios were available in the high-reward bowl while the low-reward bowl contained 3 inaccessible Cheerios. For a low-reward trial, there was 1 accessible Cheerio (with two mesh covered inaccessible Cheerios) in the low-reward bowl, and all 3 Cheerios were inaccessible in the high-rewarded bowl.

A tactile cue lining the PVC tunnel and goal box indicated which bowl had an available reward; one of two grades of silicon carbide waterproof sandpaper, coarse (P60) or fine (P1200), was paired with a specific reward type (e.g., coarse sandpaper signaled a low-reward in the black bowl on the left; Brydges et al., 2011). All sandpaper were of the same brand and were black in color. Pairings of sandpaper grade, bowl color, bowl side, and reward value were counterbalanced. All elements of the testing chamber were cleaned with 70% ethanol between each trial.

To learn the stimulus-reward associations rats were exposed to daily training sessions that consisted of 2 high-reward trials and 2 low-reward trials; the order of the 4 trials was randomized. Animals moved from the start box through the PVC tunnel into the goal box, and chose either the “correct” rewarded or “incorrect” unrewarded bowl. A choice was defined as a rat moving its nose or paw inside the bowl or touching the outside of the bowl with its nose or paw. If an animal chose the rewarded bowl first, the trial was counted as correct and the rat was allowed to consume the reward. If an animal chose incorrectly it was allowed to move to the correct bowl and consume the reward during the first 5 days of training. Decision-making was measured during the first 8 trials, after the rats had consumed at least one reward in the test chamber, by timing the latency between the incorrect selection of an inaccessible reward and the switch to choose the rewarded bowl. Starting the 6th day of training, the rat was removed immediately if it chose the incorrect side.

A learning criterion was set at 3 out of 4 trials with a correct first bowl choice for 4 out of 5 days. The number of days to reach the learning criterion was evaluated to determine if stress during adolescence impacts adult associative learning (Hammond et al., 2009). In both the stressed and control group 2 rats did not pass the learning criterion. After passing the criterion, probe trials were conducted where ambiguous/intermediate grade silicon carbide waterproof sandpaper (P220) was placed in the PVC tube connecting the start and goal boxes. On each day of probe testing a total of 5 trials were run; in addition to the 4 standard trials, one probe trial using ambiguous sandpaper was randomly inserted into the normal sequence, but the last trial was never a probe trial. A total of 5 probe trials were run over 5 days following the same design as Brydges et al. (2011). During probe testing all animals maintained the learning criterion.

After choosing a bowl during the ambiguous probe trial, the bowl choice was noted as either a high or a low-reward categorization of the ambiguous sandpaper cue and the animal was allowed to consume the reward. A number of studies using unrewarded probe trials found that animal stop responding during repeated probe trials, interpreted as a consequence of the animals learning that probes are not reinforced (Bateson and Matheson, 2007; Brilot et al., 2010; Doyle et al., 2010). To circumvent this, in the current study both high and low-rewards were present during probe trials to avoid cessation of response. A potential limitation of this design is that an initial probe interpretation may be reinforced if an animal encounters an expected reward, however, our data do not support this as all but 2 rats sampled both bowls during the 5 probe trials.

### Sucrose preference

To assess motivation to consume sucrose solution, a 24-h test for preference of 2% sucrose solution (weight/volume) relative to water was administered both prior to and after stress exposure (at 27 and 211 days of age) to determine whether preference changed over time (Strekalova et al., 2004). Animals had simultaneous access to 2 bottles placed side-by-side on the lid of their home cage: one with water and another with a 2% sucrose solution. To avoid effects from side preference, the location of the sucrose and water bottles was counterbalanced and halfway through the test, the sides of the two solutions were switched. Bottle weights were obtained before and after the 24-h period of unlimited access and relative preference was calculated: Sucrose preference = 100 × sucrose solution intake(g)/[sucrose solution intake(g) + water intake(g)]. To assess individual preferences, animals were singly-housed during the assay. To minimize social stress during testing, the individual cages of separated pairs were positioned next to each other and all rats had access to a 7.6 cm diameter PVC tube and a 2.5 × 2.5 × 8 cm pine block for enrichment (Odberg, 1987; Gross et al., 2012). Following the sucrose preference test, pairs were recombined in a clean home cage.

### Exploratory behavior

Rats were given two tests to assess exploratory behavior, an open field and a novel object test. Both tests were administered at two time points, one before and one after stress exposure (Van Dijken et al., 1992; Cavigelli et al., 2009). To minimize the potential for the second set of behavioral tasks to be influenced by the first, the two test iterations were separated by 55 days and new stimulus objects were used during each novel object test. All tests were run in a 122 × 122 × 46 cm opaque Plexiglas arena. Each task involved 5 min of free exploration during which latency to leave a 7.6 cm diameter PVC tube shelter was measured. All animals started both exploratory tests inside the PVC tube shelter; the tube was placed along the base of one arena wall in the same position and orientation for all tests.

#### Exploratory task 1: open field

To compare activity levels between adolescent-stress and control animals, activity in the arena was quantified with a video-recorded open field assay at two time points (28 and 84 days of age, pre and post chronic unpredictable stress). During video analysis an 8 × 8 grid was used to quantify activity by counting the number of squares crossed on the grid. Crossing of grid squares along the walls of the arena and the proportion of time spent in squares along the arena walls were quantified as indicators of thigmotaxis, a positive correlate of anxiety (Simon et al., 1994).

#### Exploratory task 2: novel object

Response to novelty was evaluated before and after chronic unpredicatable stress (at 29 and 85 days of age) with two behavioral measures: time to leave the PVC shelter (i.e., when all 4 feet were touching the arena floor) and latencies to physically contact the two novel objects in the arena with either a paw or nose. The novel objects varied in texture, color, and size. Several plastic objects were used including a translucent red triangle, an opaque matt yellow bowl, a shiny yellow cylinder, and a translucent shelter.

### Data analysis

Sucrose preference and cognitive bias data conformed to the assumptions for parametric analyses. SNC data were square root transformed to achieve normality. Exploratory behavioral data from the novel object and open field assays were natural log transformed to achieve normality. To test whether chronic unpredictable stress during adolescence affected sucrose preference or exploratory behavior over time (pre and post chronic unpredictable stress), we used 2 factor (time and stress condition) repeated measures ANOVAs to compare across the 2 tests. For *post-hoc* analysis, independent samples two-tailed *t*-tests were used to compare the stress and control groups within the 2 test iterations. Only significant *post-hoc* findings are reported.

To evaluate ambiguity interpretations in the cognitive bias assay, we tested the first two ambiguous probe exposures separately using univariate general linear models because response to the ambiguous probe changes with repeated exposures; initial exposures are more reliable measures of affect (Bateson and Matheson, 2007; Brilot et al., 2010; Doyle et al., 2010). Following individual analysis of the first and second probes, all 5 probe exposures were evaluated with a repeated measures general linear model as in Brydges et al. (2011) and Burman et al. (2009). To determine whether activity or motivation to consume a reward impacted performance in the cognitive bias test, we included activity in the open field and sucrose preference as covariates in the ambiguous probe general linear models. Neither activity nor sucrose preference were significant factors in explaining variation in the data, so they were removed from the model [activity: *F*_(1, 12)_ = 0.929, *P* = 0.36; sucrose preference: *F*_(1, 12)_ = 0.590, *P* = 0.46]. The associative learning and decision-making data were analyzed with independent samples two-tailed *t*-tests. To determine whether animals that experienced adolescent stress had a stronger response to reward devaluation than control animals, repeated measures ANOVAs were used to assess behavior for pre-shift days 4–12 and post-shift days 13–18. To assess the impacts of activity and motivation to consume a reward on SNC scores locomotion in the open field and sucrose preference were included as covariates in the repeated measures ANOVAs; neither factor significantly explained variation in the data and were subsequently removed from the model [Pre-shift: activity: *F*_(1, 12)_ = 0.065, *P* = 0.81; sucrose pref: *F*_(1, 12)_ = 0.092, *P* = 0.77; Post-shift: activity: *F*_(1, 12)_ = 1.959, *P* = 0.20; sucrose pref: *F*_(1, 12)_ = 1.984, *P* = 0.20]. To evaluate response to the reward devaluation, lick numbers on the first day of post-shift were subtracted from the average of the last 3 days of pre-shift. These difference scores were tested with a two-tailed *t*-test. Analyses were run in SPSS; values are reported as means ± standard deviation.

## Results

### Successive negative contrast

**Figure 4:**
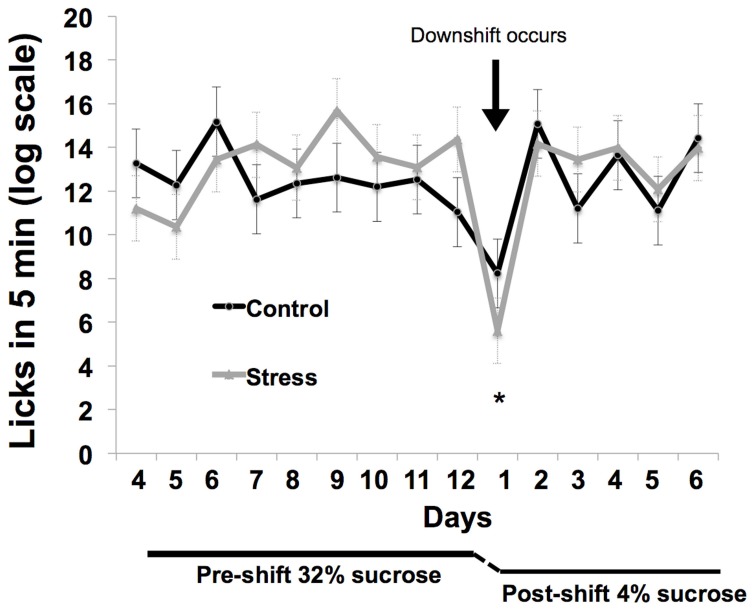
**Coping response in a successive negative contrast test following stress during adolescence**. Animals exposed to chronic unpredictable stress during adolescence exhibited a long-term increase in reward loss sensitivity as measured by successive negative contrast (SNC). The asterisk indicates a significant difference in the downshift scores between the adolescent-stressed and control animals.

Response to the reward downshift was greater in the adolescent-stress group than in the control animals [*T*_(1, 14)_ = 2.216, *P* = 0.04, *d* = 1.02, see Figure 4]. No differences were found in the pre-shift lick rates of the adolescent-stress and control animals [RM ANOVA, *F*_(1, 6)_ = 0.092, *P* = 0.77] or over time [*F*_(1, 6)_ = 4.022, *P* = 0.37], nor was there an interaction [*F*_(1, 6)_ = 1.173, *P* = 0.61]. Post-shift lick rates changed over time as rats returned to pre-shift licking rates [RM ANOVA, *F*_(1, 6)_ = 9.911, *P* = 0.01], but stress and control animals did not differ in their post-shift lick rates [RM ANOVA, *F*_(1, 6)_ = 0.003, *P* = 0.95], nor was there an interaction [*F*_(1, 6)_ = 0.644, *P* = 0.70].

### Cognitive bias assay

**Figure 5:**
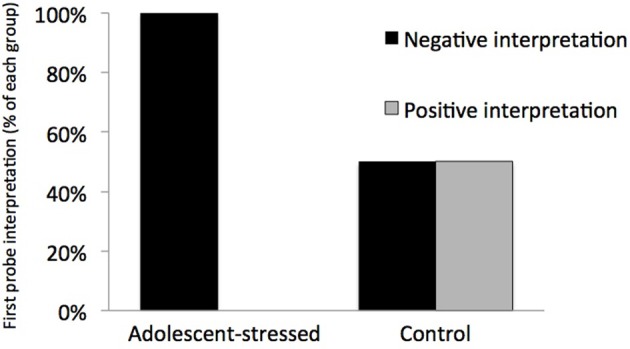
**Interpretation of a novel ambiguous stimulus on the first day of an ambiguous judgment test for cognitive bias**. All animals that experienced stress during adolescence interpreted the ambiguous cue as negative, indicating a long-term negative cognitive bias, while control animals were equally likely to interpret the cue as positive or negative.

**Figure 6:**
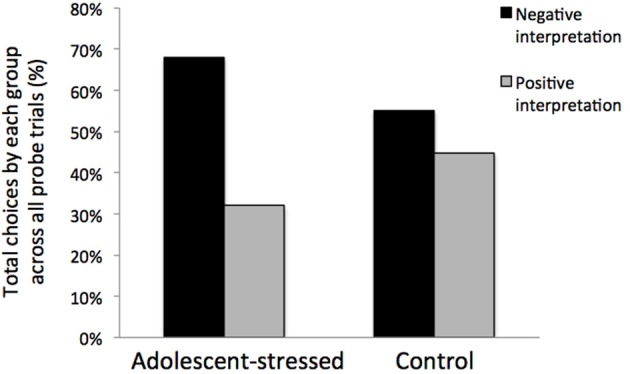
**Interpretation of an ambiguous stimulus during all cognitive bias testing days**. Rats that experienced stress during adolescence trended toward a negative cognitive bias across the 5 day testing period.

**Figure 7:**
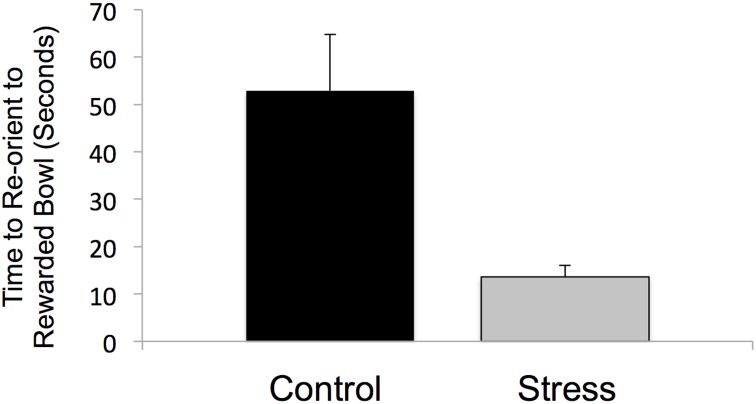
**Latency to move to the correct, rewarded bowl after the first incorrect choice**. At the first encounter with the unrewarded bowl during cognitive bias training, prior to associative learning, animals that experienced stress during adolescence had a shorter latency to correct their choice and reorient to the rewarded bowl.

All stressed animals interpreted the ambiguous cue as negative on the first day of probe testing, demonstrating a negative cognitive bias that differed from the control animals whose interpretations were half positive and half negative [*F*_(1, 12)_ = 5.000, *P* < 0.05, *R*^2^ = 0.33, see Figure 5]. This difference in the interpretation of the ambiguous probe was not significant in subsequent trials [Day 2: *F*_(1, 12)_ = 0.000, *P* = 1.00; GLM 5 days: *F*_(1, 12)_ = 0.471, *P* = 0.508]. The total number of positive and negative probe interpretations from each group are depicted in Figure 6. Within the first two days of training, adolescent-stressed animals were faster to correct wrong decisions by abandoning the wrong bowl, reorienting, and choosing the correct bowl [*t*-test *T*_(1, 14)_ = 3.245, *P* = 0.01, *d* = 1.62, see Figure 7]. However, animals stressed during adolescence showed no difference in the number of days to learn the associative task compared with controls [*t*-test, stress: 26 ± 3 vs. control: 25 ± 5; *T*(1, 10) = 0.419, *P* = 0.68].

### Sucrose preference

Sucrose preference decreased over time [RM ANOVA effect of time: *F*_(1, 7)_ = 5.680, *P* = 0.04], but there was no effect of stress [RM ANOVA effect of stress: *F*_(1, 7)_ = 0.417, *P* = 0.53] and no interaction between stress condition and time [RM ANOVA stress × time interaction: *F*_(1, 7)_ = 0.631, *P* = 0.42; time 1: stress 83 ± 7% vs. control 82 ± 7%; time 2: stress 78 ± 7% vs. control 74 ± 8%].

### Open field activity scores

Activity increased over time, which is consistent with previous studies indicating that age influences exploratory behavior in an open field [RM ANOVA effect of time: *F*_(1, 7)_ = 8.454, *P* = 0.01; Bronstein, 1972]. There was no effect of stress [RM ANOVA effect of stress: *F*_(1, 7)_ = 0.093, *P* = 0.77], nor was there an interaction between stress condition and time [RM ANOVA stress × time interaction: *F*_(1, 7)_ = 1.423, *P* = 0.25; time 1, stress 244 ± 30 squares crossed vs. control 233 ± 86 squares crossed; time 2, stress 282 ± 29 squares crossed vs. control 303 ± 40 squares crossed].

Thigmotaxis decreased over time in all animals [RM ANOVA effect of time: *F*_(1, 7)_ = 97.685, *P* < 0.00]. There was no effect of stress condition [RM ANOVA effect of stress: *F*_(1, 7)_ = 0.110, *P* = 0.75], and no interaction between stress and time [RM ANOVA stress × time interaction: *F*_(1, 7)_ = 0.359, *P* = 0.56; time 1, stress 283 ± 12(s) vs. control 282 ± 10(s); time 2, stress 233 ± 15(s) vs. control 238 ± 21(s)].

### Novel object

The latency to approach a novel object decreased in animals exposed to stress during adolescence [RM ANOVA effect of stress: *F*_(1, 7)_ = 4.682, *P* < 0.05]. In the second test iteration rats exposed to adolescent-stress were faster to approach a novel object than control animals [latency to approach novel object at time 2, stress: 4.6 ± 2(s) vs. control: 16 ± 13(s); *T*_(1, 14)_ = 2.419, *P* = 0.03, *d* = 1.23] with no baseline difference in the approach latency prior to stress exposure [latency to approach novel object at time 1, stress: 15.4 ± 19(s) vs. control: 16.7 ± 13(s); *T*_(1, 14)_ = 0.156, *P* = 0.88]. While this difference appears to be a real biological effect, the variance between the groups was high which may explain the lack of interaction between time and treatment [RM ANOVA stress × time interaction: *F*_(1, 7)_ = 1.544, *P* = 0.23].

Latency to leave the PVC shelter decreased over time in all animals, which is congruous with previous findings that behavior in a novel object test changes as animals reach adulthood [RM ANOVA effect of time: *F*_(1, 7)_ = 11.179, *P* = 0.01; Saul et al., 2012]. During the novel object test, 15 days after the completion of the chronic unpredictable stress treatment, rats exposed to stress during adolescence left the PVC shelter faster than control animals [exit latency at time 2, stress: 2.3 ± 0.6(s) vs. control: 7.3 ± 1.8(s); *T*_(1, 14)_ = 2.240, *P* = 0.04, *d* = 3.73]. There was no baseline difference in the latency to leave the PVC shelter prior to stress exposure [exit latency at time 1, stress: 2 ± 0.5(s) vs. control: 1.7 ± 1(s); *T*_(1, 14)_ = 0.344, *P* = 0.74].

## Discussion

Our results show that chronic unpredictable stress during adolescence has long-term effects on coping response, cognitive bias, and decision-making. Associative learning and sucrose preference, however, were not affected by stress exposure during adolescence. The novel object test showed increased boldness behaviors 15 days after completion of the chronic unpredictable stress paradigm. Activity and thigmotaxis in the open field were not affected by prior adverse experience. Stress-exposed rats were faster to leave a familiar shelter in an environment containing novelty and approached novel objects more quickly than control animals. The successive negative contrast test demonstrated that stress during adolescence induces a stronger response to the devaluation of an expected reward in adulthood. The sucrose preference test demonstrated that stress during adolescence does not alter motivation to consume a reward, confirming that the altered response to reward devaluation exhibited by animals exposed to stress during adolescence was not due to a difference in reward salience, but was a reaction to the downshift in reward value.

Exposure to stress during adolescence also decreased the latency to correct a choice and locate a food reward after an incorrect decision. In an early phase of training for the cognitive bias assay, adolescent-stressed animals were faster at abandoning, reorienting, and switching their choice of food bowl after encountering a bowl with an inaccessible reward than the control animals. The results from the sucrose preference test exclude the possibility that the shorter latency to find the reward after an incorrect decision is due to a difference in motivation to obtain the reward, as the preference test demonstrates that motivation to consume a reward is unchanged by stress during adolescence. Thus, the expediency of decision-making in stressed animals could be the result of decreased behavioral inhibition or increased impulsivity when compared to the control animals. Animals exposed to exogenous corticosterone during adolescence show a form of impulsivity marked by an increased preference for an immediate, small reward rather than a larger reward delivered after a variable delay (Torregrossa et al., 2012). It is possible that the decreased latency to abandon a first choice and transition to a second choice demonstrated by adolescent-stress animals also reflects increased impulsivity. Long-term changes in impulsivity behaviors may be underpinned by stress-induced changes in the brain. Stress may impair maturation processes that typically occur during adolescence, such as myelination in the prefrontal cortex, thereby prolonging an immature-like state of top-down connectivity into adulthood (McCormick, 2007). An immature-like state in prefrontal cortex could maintain increased impulsivity behaviors characteristic of the adolescent stage, and alter behavioral inhibition and decision-making in adulthood.

Our results showed that stress during adolescence induces a long-term negative cognitive bias. This finding, along with the SNC results demonstrating increased sensitivity to reward loss, indicate that stress during adolescence generates a long-term negative background emotional state (Burman et al., 2008; Mendl et al., 2009). A negative background emotional state can bias decision-making and expectations for the future; humans with a negative emotional state exhibit biases in attention (e.g., greater attention to threatening stimuli), memory (e.g., enhanced negative memory retrieval), and judgment (e.g., risk and ambiguity aversion, Paul et al., 2005). It is important, however, to keep in mind the potential ecological context of a negative cognitive bias induced by stress. For example, in sites of high predation, traits like threat bias and risk aversion may serve an adaptive function. If threat is prevalent in an environment, it may be advantageous to more readily treat ambiguity as negative or a potential threat (Mendl et al., 2009). Stress induced programming during adolescence for a long-term threat bias may serve to prepare an individual to cope with future exposure to a dangerous environment. Human studies suggest that the consequences of a negative cognitive bias are far reaching, but the full impacts of a negative cognitive bias in non-human animals are not yet clear (Winkielman et al., 2007).

The ambiguous judgment cognitive bias task used here captured differences in the interpretation of ambiguity as a result of stress during adolescence. The results of the 5 probe trials evaluated together, however, highlight a limitation of the ambiguous judgment test. During the first exposure to the ambiguous probe, animals interpret the novel ambiguous stimulus based only on their own biases and life history, whereas subsequent exposures to the ambiguous probe are influenced by previous interpretations of the probe. Thus, repeated probe tests can be subject to effects from learning (Doyle et al., 2010). In the current study the initial ambiguous probe trials were analyzed separately from subsequent probe trials similar to Brilot et al. (2010). Future studies that use the ambiguous judgment task should analyze initial probe exposures separately, as the use of repeated probe tests allows for learning and can yield misleading results (Brilot et al., 2010; Doyle et al., 2010).

The current study found that sensitivity to reward loss in adulthood is intensified by exposure to stress during adolescence, suggesting that animals exposed to adverse events during this period can undergo a long-term change in coping with challenge. This result could help explain an interesting phenomenon documented in previous studies: adolescent-stressed animals can appear to have unaltered behavior, temperament, and learning in adulthood, until they encounter a challenge, at which point behavioral differences become apparent (Watt et al., 2009; Vidal et al., 2011; McCormick et al., 2012). Our results suggest that the altered response to challenge demonstrated by adult animals exposed to stress during adolescence could arise from a long-term change in coping response that has behavioral and cognitive consequences that only become apparent upon subsequent exposure to stress.

Immediately following exposure to isolation and unpredictable housing during adolescence, exploratory behavior in an elevated plus maze is increased, however, a month following stress exposure exploratory behavior is decreased relative to controls (McCormick et al., 2008). Our results expand upon this finding to demonstrate that 2 weeks following physical and social stress during adolescence, male rats are faster to approach novelty, suggesting increased exploratory behavior. The contrast in effects of closely related stress paradigms emphasizes the need for longitudinal studies that evaluate the consequences of specific stress paradigms and span multiple life stages in order to more completely understand how resilience and vulnerability to stress change over the lifetime of an organism.

### Conflict of interest statement

The authors declare that the research was conducted in the absence of any commercial or financial relationships that could be construed as a potential conflict of interest.
